# Supplementary material for: Multidimensional Functional Profiling of Human Neuropathogenic FOXG1 Alleles in Primary Cultures of Murine Pallial Precursors
Source: Int J Mol Sci. 2022 Jan 25;23(3):1343. doi: 10.3390/ijms23031343 (PMC8835715; doi:10.3390/ijms23031343)
Supplement: Supplementary file 1 [file ijms-23-01343-s001.zip › ijms-1555754-supplementary.pdf]

Multidimensional functional profiling of human neuropathogenic FOXG1 alleles in primary cultures of murine pallial precursors,
by Frisari et al.

SUPPLEMENTARY INFORMATION

It includes:

- Figures S1-S5
- Tables S1-S2

Figure S1

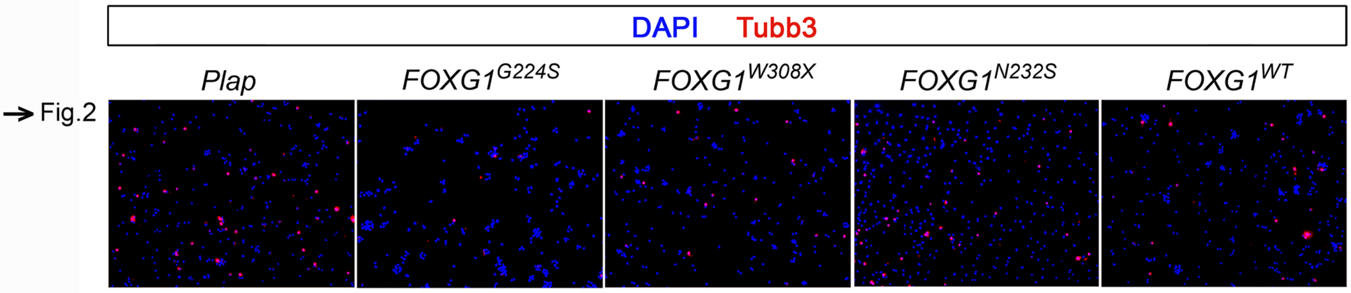

Figure S1. Examples of primary data referred to in Figure 2.

Figure S2

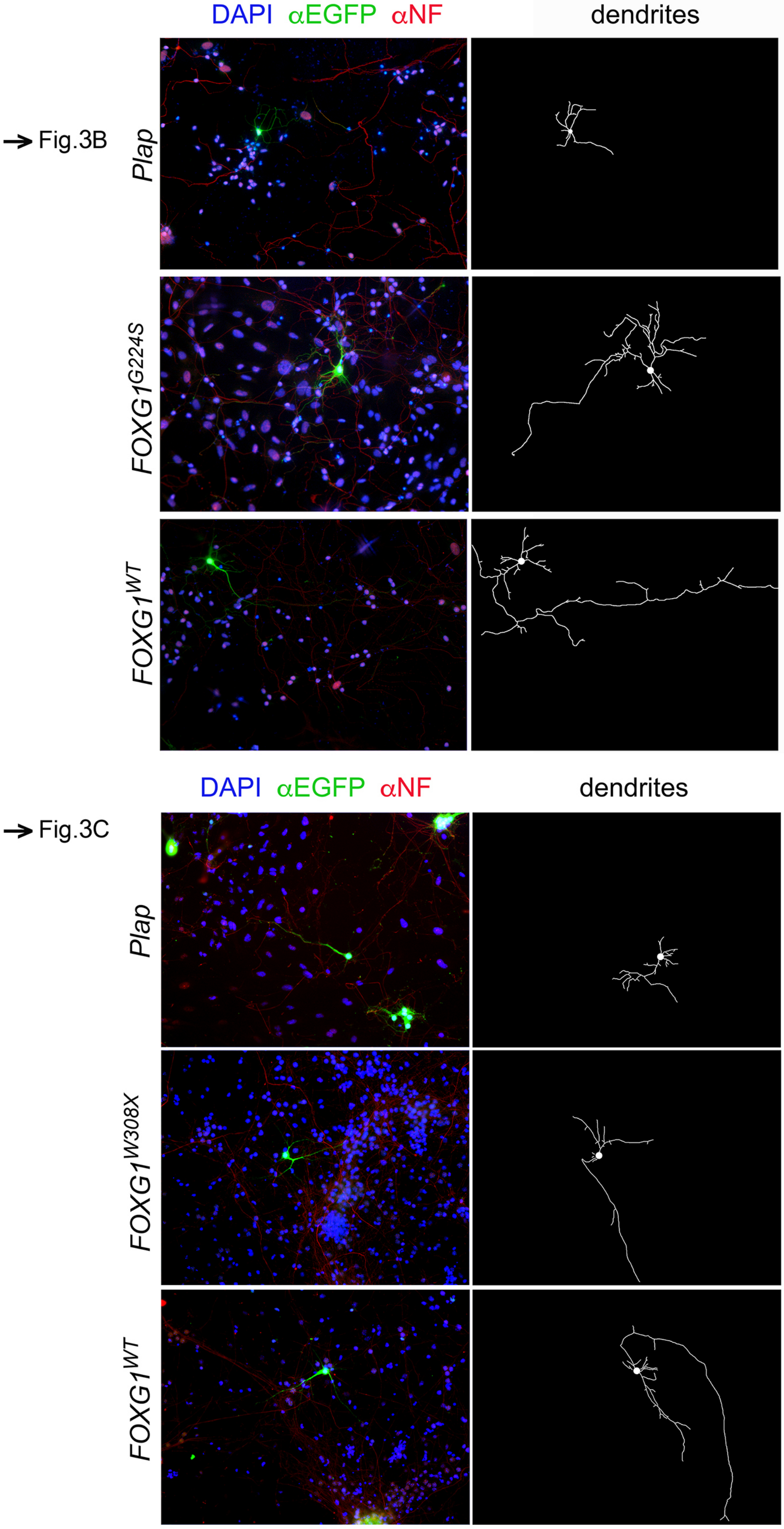

Figure S2. Examples of primary data referred to in Figure 3.

Figure S3

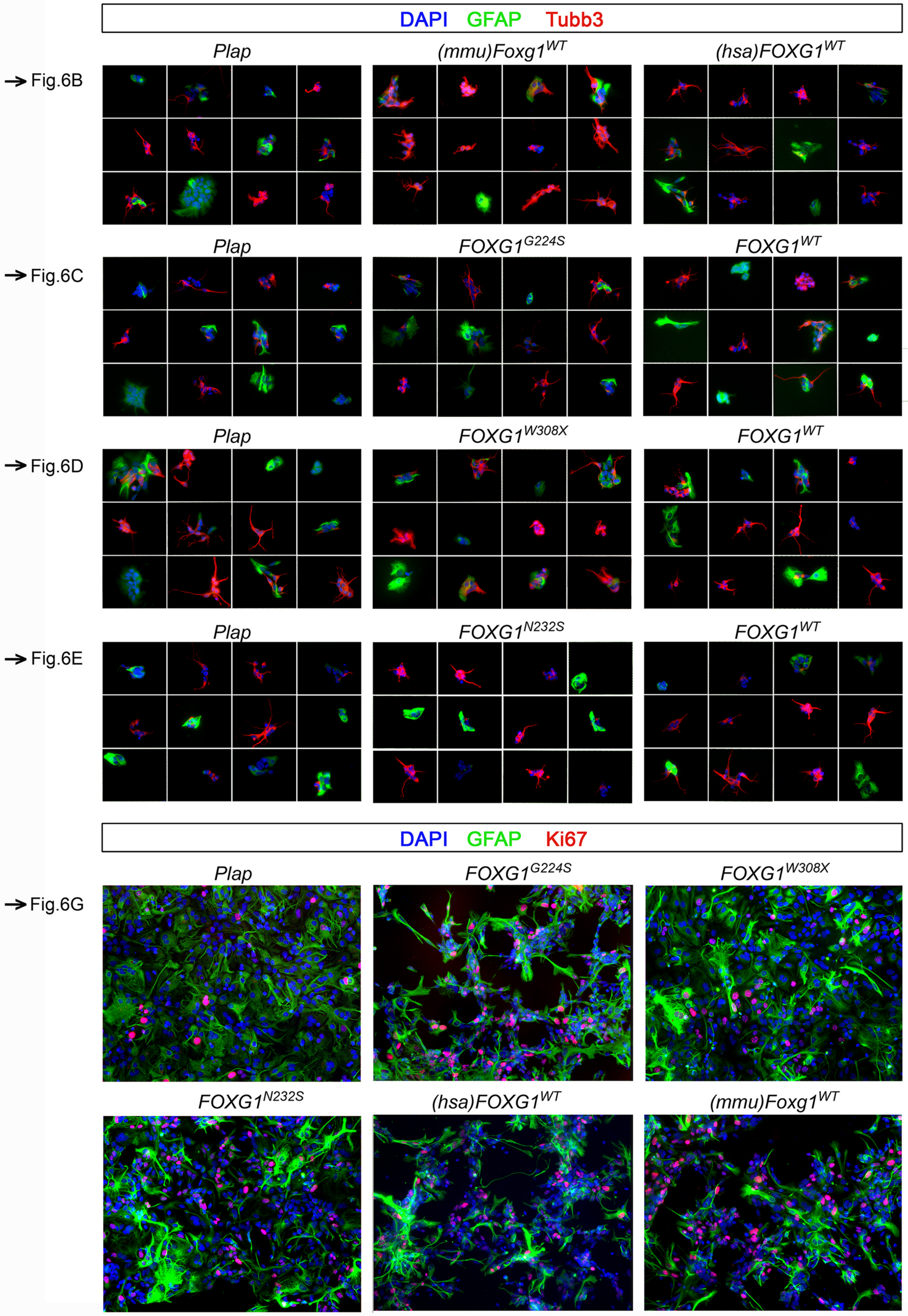

Figure S3. Examples of primary data referred to in Figure 6.

Figure S4

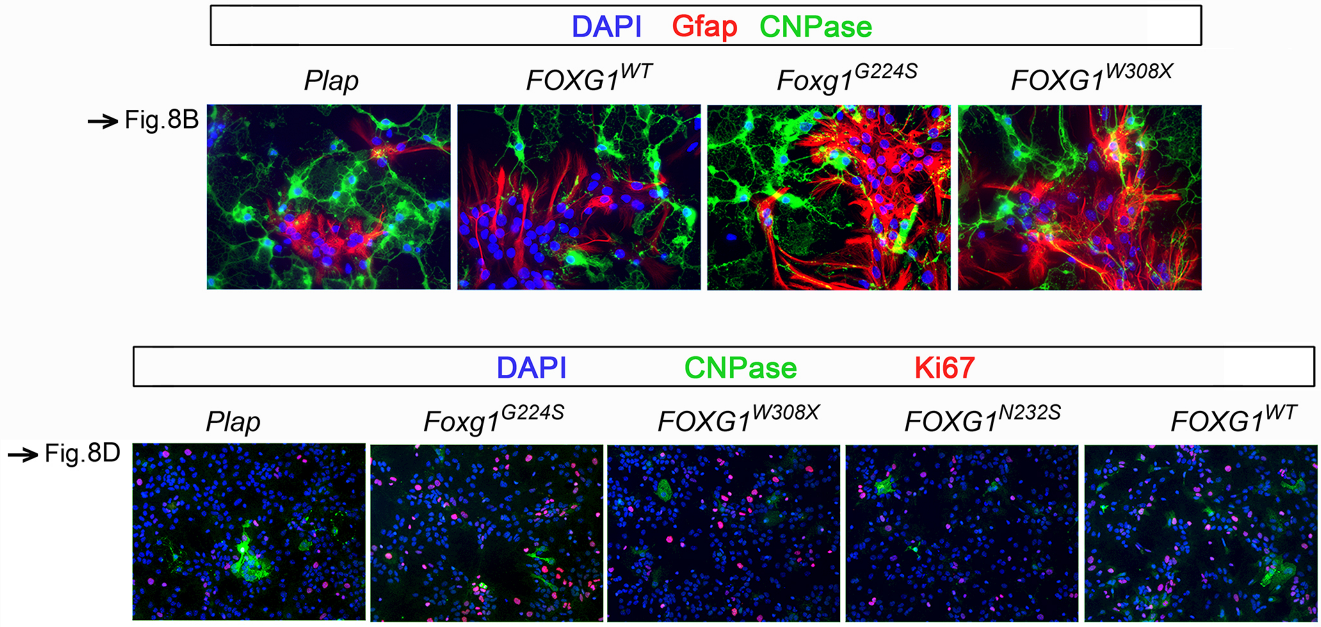

Figure S4. Examples of primary data referred to in Figure 8.

Figure S5

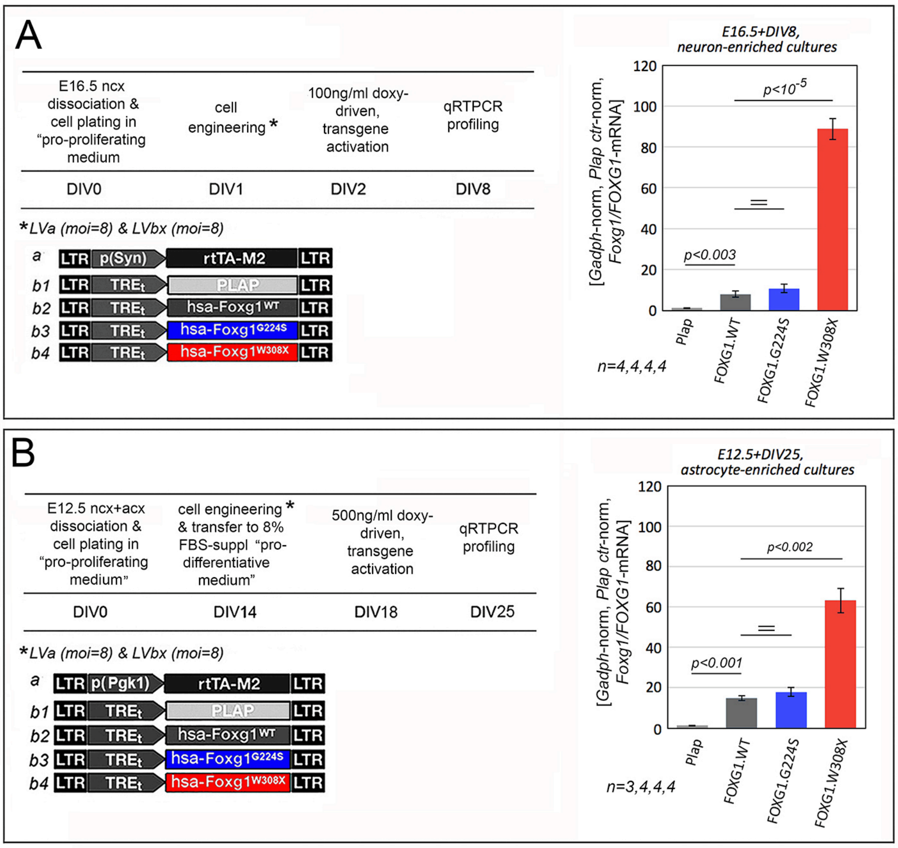

Figure S5. Cumulative Foxg1/FOXG1-mRNA levels in engineered neural cultures referred to in Figures 5 and 7.

Table S1. Oligonucleotides list.

mmuHes1_3UTR/F: 5’ CAC TGC TAC CCG TAA AGT CCC TAG CC 3’
mmuHes1_3UTR/R: 5’ TGG TCA GTC ACT TAA TAC AGC TCT CTA C 3’
Arc/F: 5’ CCC TCA TCT GTC TGC CCT GG 3’
Arc/R: 5’ ACC CAA AGA GCC CTG GAC AC 3’
Cacna2d2/F: 5’ CGT ACC CAT CAG GAC CAA CC 3’
Cacna2d2/R: 5’ CCT GGT CCC ACT GAT GGT TC 3’
Gad1/F: 5’ ACC AGT TGC TGG AAG GCA TGG AAG G 3’
Gad1/R: 5’ AGA GCT GGT TGA AAA ATC GAG GGT GAC 3’
Gad2/F: 5’ ATA ATT GGG AAT TGG CAG ACC AAC CGC 3’
Gad2/R: 5’ CTG CTG CTA ATC CAA CCA TAT CCA ATC C 3’
Grik3/F: 5’ GTC GCC CAT CGA CTC CGC TGA TG 3’
Grik3/R: 5’ TCA GCT GTG AGC GTC CGC TGG AT 3’
Grik4/F: 5’ CGA GCT TTG TCT ACC CGC TGT GTC A 3’
Grik4/R: 5’ GTC AGC CAG GTC ATC CAC AGA CTC AA 3’
Grin2A/F: 5’ CTC ACC CTG GAA GAG GCA GAT TGA C 3’
Grin2A/R: 5’ AGC CAG CAT GTA GAA AAC TCC TGC CA 3’
Grin2c/F: 5’ CTG GAT TTC GTC TCC TCT CAG ACC C 3’
Grin2c/R: 5’ GTA TTC CTC CAG CAC CTT GAA CAG CAC 3’
Foxg1.5'utr/F: 5' TAG AAG CTG AAG AGG AGG TGG AGT GC 3'
Foxg1.5'utr/R: 5' CAG ACC CAA ACA GTC CCG AAA TAA AGC 3'
Foxg1.cds/F: 5' CGA CCC TGC CCT GTG AGT CTT TAA G 3'
Foxg1.cds/R: 5'GGG TTG GAA GAA GAC CCC TGA TTT TGA TG 3'
Scn11a/F: 5’ AGC GCC TTG TTT TCT CGG TA 3’
Scn11a/R: 5’ CCC ACG ATG ACC TTC AGA CC 3’
mGapdh5/F: 5’ ATC TTC TTG TGC AGT GCC AGC CTC GTC 3’
mGapdh5/R: 5’ GAA CAT GTA GAC CAT GTA GTT GAG GTC AAT GAA GG 3’
mmuGabra1/F: 5' AAA CCA GTA TGA CCT TCT TGG ACA AAC AGT TGA C3'
mmuGabra1/R: 5' GTG GAA GTG AGT CGT CAT AAC CAC ATA TTC TC 3'
Npas4/F: 5’ CTC CGC CAG CAG CTA AAC T 3’
Npas4/R: 5’ CAG GGA TGG TGT AAA GAG TGG A 3’
Kcnk2/F: 5’ TAC AGA CTG CTG GCA TAG GTC GTC TC3’
Kcnk2/R: 5’ CAG TAG ATG TAA GTA CGG GCA CAG AGG 3’
Glt-1/F: 5’ CTC CCT CTT ATC ATC TCC AGT TTA ATC ACA G 3’
Glt-1/R: 5’ AAC ACC AGG ATG ACC CCC AGC ACG 3’
Kir4.1/F: 5’ CGA TTT ATC AGA GCA GCC ACT TCA CCT TC 3’
Kir4.1/R: 5’ CGT TTG TCA GCA ATG TGC TCC ATT CTC AC 3'
Connexin43/F: 5' TCA GCC TCC AAG GAG TTC CAC CAC TTT 3'
Connexin43/R: 5' AGG AGC AGG ATT CTG AAA ATG AAG AGC ACC 3'
Adk/F: 5' GAT CAA AAC CAG GAA GAG ATC ATT GAC ACC A 3'
Adk/R: 5' GTC TGG CTT CTC GGG AAA GGT ACA G 3'
mGlur5/F: 5' ACG CTT GGG TCT AGC AGG GGA ATT TCT A 3'
mGlur5/R: 5'CCT GAT GTC AAG TGG TTT GAT GAT TAT TAT CTG AA 3’

Table S2. Primary antibodies list

anti-GFP, chicken polyclonal (Abcam, ab13970), used at 1:500
anti-Tubb3, mouse monoclonal (clone Tuj1, Covance, MMS-435P), used at 1:1000
anti-NF, mouse monoclonal (Abcam, ab7795), used at 1:400
anti CNPase, mouse monoclonal (clone 11-5B, Abcam ab6319), used at 1:400
anti CNPase, rabbit polyclonal (Invitrogen, PA5-27972), used at 1:400
anti-GFAP rabbit polyclonal (DAKO #Z0334), used at at 1:400
anti-Ki67, mouse monoclonal (BD Pharmingen #550609), used at at 1:50
